# A solitary hemangioblastoma of the posterior brain fossa: the role of radiotherapy

**DOI:** 10.11604/pamj.2020.36.114.22282

**Published:** 2020-06-19

**Authors:** Fatima Zahra Abboud, Moulay Ali Youssoufi, Touria Bouhafa, Khalid Hassouni

**Affiliations:** 1 Department of Radiation Oncology, University Hassan II, Hospital of Fez, Fez, Morocco,; 2 Medical Physics Unit, Oncology Hospital, University Hospital Hassan II, Fez, Morocco

**Keywords:** Hemangioblastoma, radiotherapy, posterior brain fossa, surgical excision

## Abstract

We report here the case of a patient admitted for management of posterior fossa cerebral hemangioblastoma. A 16-year-old male patient with a history of intracranial hypertension syndrome consisting of progressively worsening headache, vomiting, especially morning and jet vomiting, and decreased visual acuity. The patient's symptomatology worsened a few days later with the appearance of a disturbance of balance with enlargement of the sustentation polygon. The patient initially benefited from a brain computed tomography (CT) scan that objectified a solidocystic process of the posterior brain fossa. The patient then underwent a surgical excision that was considered partial and the diagnosis of hemangioblastoma was made on the surgical specimen. Since the surgical removal was partial the patient was referred to our training where he received external radiotherapy on his hemangioblastoma of the posterior brain fossa. The patient was examined one month after the end of irradiation; he presented a spectacular improvement in his neurological symptomatology with a clear regression of balance disorders. The standard treatment for cerebellar hemangioblastoma is complete microsurgical removal, but our results show a high level of efficacy for fractional photon radiotherapy after partial surgery of this benign tumour.

## Introduction

Hemangioblastomas (HB) are benign tumors that comprise 2% of all intracranial tumors and 3-4% of all spinal tumors [1]. They are commonly associated with Von Hippel-Lindau syndrome (VHL). HB have been documented throughout the central nervous system, but the cerebellum and spinal cord are the most common locations. HB account for 7-12% of all infratentorial tumors in adults [2]. Because hemangioblastomas are benign in nature, the standard treatment is complete microsurgical removal [3]. We report a patient with a solitary HB of the posterior brain fossa (PBF), who received a partial surgical exeresis followed by an external three-dimensional conformal radiotherapy (3D RT) with an improvement of his symptomatology from a clinical point of view. From a radiological point of view, the control cerebral magnetic resonance imaging (MRI) at 3 months after the end of the irradiation showed a decrease in the size of the tumoral process of the PBF.

## Patient and observation

**Figure 1:**
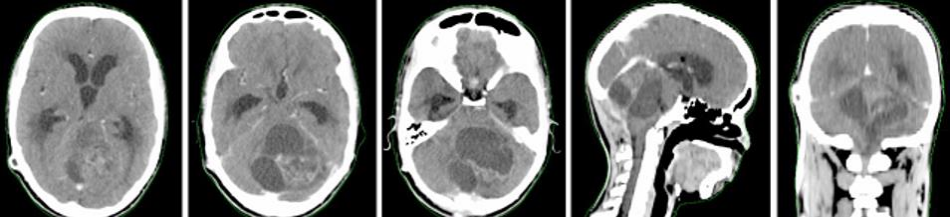
cerebral CT sections with contrast product injection in all three planes, showing the cerebellar tumour process with triventricular hydrocephalus

**Figure 2:**
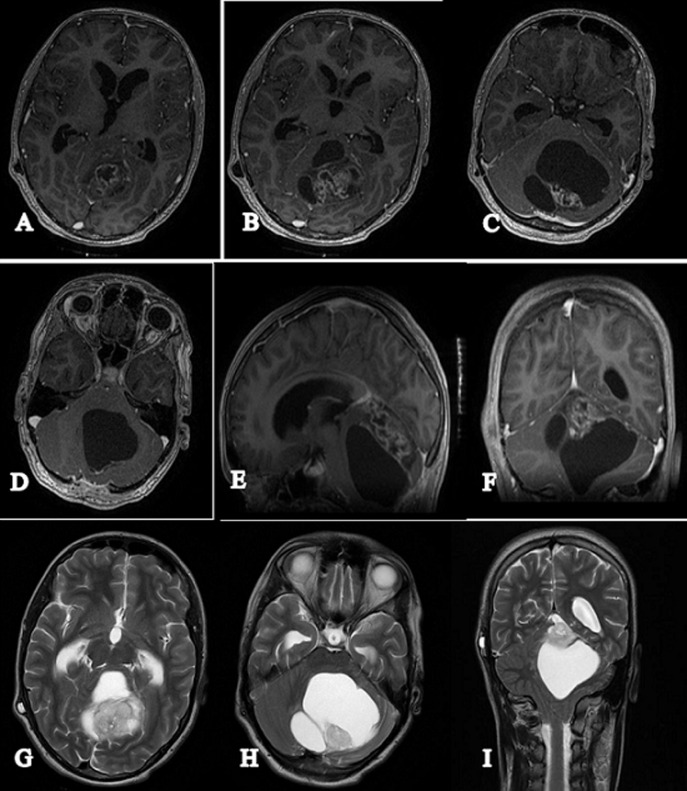
axial section brain MRI T1 injected (A+B+C+D) sagittal slice (E) and coronal slice (F), axial sections T2 (G+H) and coronal slice (I), showing cerebellar hemangioblastoma with both fleshy and cystic components

**Figure 3:**
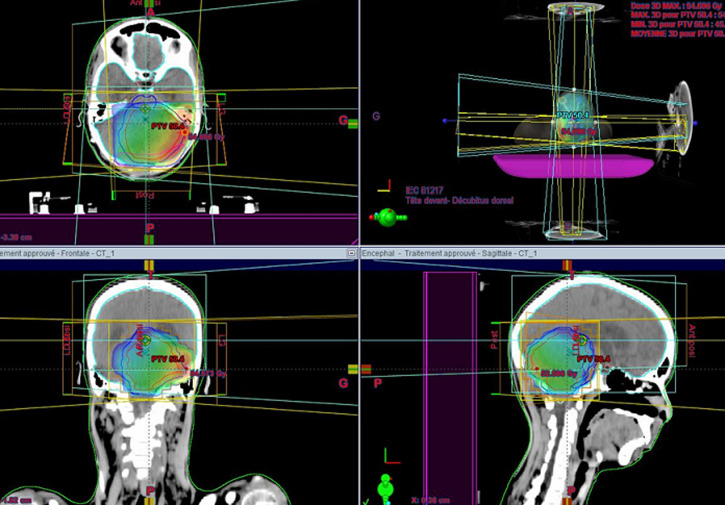
disposition of irradiation beams on the three plans (axial, sagittal and coronal)

**Figure 4:**
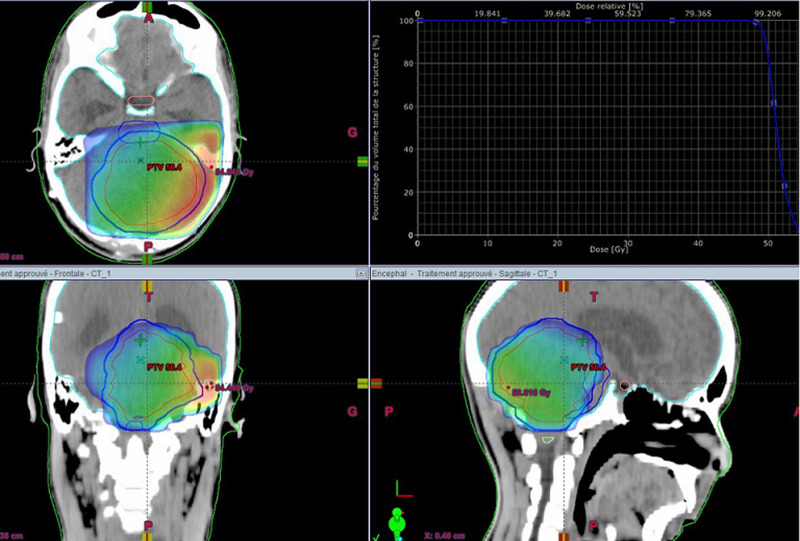
three plans view of the conformity of the prescribed dose to the target volume in blue and the respect of the optic chiasm and brainstem

**Figure 5:**
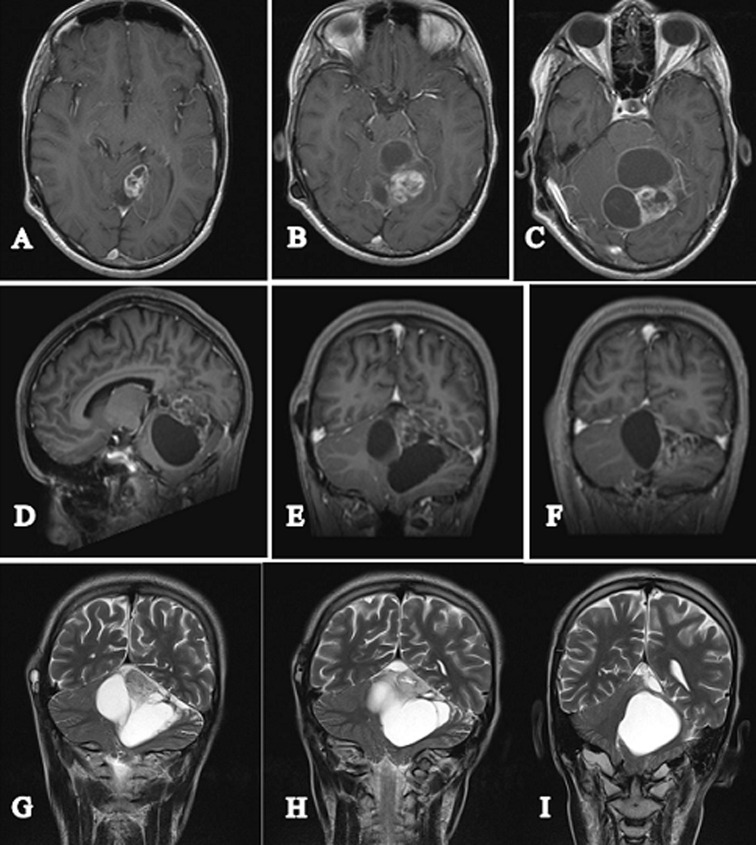
brain MRI control showing a decrease in the size of the tumor process after radiation therapy; axial section T1 injected (A+B+C) sagittal slice (D) and coronal sliceS (E+F), coronal sections T2 (G+H+I)

A 16-year-old male patient who presented, 2 months before his consultation, with rapidly progressive worsening headaches that became increasingly resistant to the usual analgesics, These headaches were accompanied by nausea, morning jet vomiting and decreased visual acuity. Several days later, disturbances of balance appeared in the patient. He denied any notion of fever, fatigue, altered general condition or seizures. At the time of questioning there was no notion of a similar case among first-degree relatives of the patient or other family members. Physical examination on admission found a conscious patient with a Glasgow score of 15/15, well-developed and well-nourished. At the neurological examination the patient presented a balance disorder with widening of the sustentation polygon, walking was impossible without help and was heeling, standing was also impossible without help, there was no sensory or motor deficit or cognitive impairment in the patient. On ophthalmological examination, the patient's visual acuity was 8/10 and the fundus was normal. Laboratory evaluations including complete blood count, electrolytes and basic metabolic profile were normal. The patient was investigated by a brain CT scan that showed a cerebellar tumour process with two components; a fleshy component and a cystic component, measuring 70 mm in diameter; this process compresses the 4th ventricle and leads to significant tri-ventricular hydrocephalus with signs of trans-ependymal resorption of cerebrospinal fluid (Figure 1). The exploration was completed by a cerebral MRI which showed a solido-cystic cerebellar tumour process measuring 70*57*60 mm evoking a pilocytic astrocytoma responsible for tonsillar involvement (Figure 2). The patient was then scheduled for surgery, but the surgical removal was partial due to the deep localization of the tumor process and the risk of hemorrhage during the operation. Anatomopathological and immunohistochemical analysis of the surgical specimen confirmed the diagnosis of hemangioblastoma. Upon diagnosis of cerebellar hemangioblastoma, the patient underwent a whole-body CT scan for pancreatic, renal or adrenal processes in Von Hippel-Lindau syndrome, but the imaging result was without detectable abnormalities, which made it impossible, in addition to the fundus which was initially normal, to talk about Von Hippel-Lindau syndrome. As the surgical removal was partial and the patient's clinical symptoms did not improve, the patient was referred to our hospital where he was given three-dimensional conformal external radiotherapy at a total dose of 50,4Gy in 28 fractions, at a daily dose fraction of 1,8Gy, one fraction per day and 5 sessions per week, over 5.6 weeks, using the 3D conformal radiotherapy technique with fields prescribed to the 100% isodose line, using 6-MV photons (Figure 3). We were able to keep the tolerable doses to the organs at risk such as the brain stem, optic chiasma, optic nerves, retina and pituitary within normal limits and at the same time deliver the expected radiation dose to the tumour site (Figure 4). The patient received the planned treatment and the tolerance was good. He was examined weekly during the treatment by our team. No significant side effects were observed, with the exception of grade I alopecia. During the first few weeks of radiotherapy, the patient was put on high-dose corticosteroid therapy and then the dose was gradually reduced until it was stopped, with good tolerance of the radiotherapy until the end of the spread. The patient was examined after one month and showed a dramatic improvement in balance with no nausea and vomiting, as well as an improvement in visual acuity. A cerebral MRI was done in the patient at 3 months after the end of irradiation which showed a decrease in the size of the cerebellar process (Figure 5).

## Discussion

The term HB was introduced in 1928 by Cushing and Bailey [4] to define vascular tumours of the nevraxis as distinct from angiomatous malforma tions. Hemangioblastomas are highly vascularized tumors and occur almost exclusively in the posterior fossa, most commonly in the cerebellum. Hemangioblastomas account for 7-12% of all infratentorial tumors in adults [2]. Subtentorial involvement is more frequent (92.6%) with cerebellar hemispheres being affected four times more often than vermis. HB may occur as isolated sporadic tumors, or as a component of VHL, an autosomal dominant inherited disorder with incomplete penetrance and expression. Up to 80% of patients affected by VHL are associated with central nervous system HB. On average a new lesion develops in patients with VHL every 2.1 years [5]. HB are vascular tumors, but spontaneous hemorrhage is rare. It was reported that the spontaneous hemorrhage risk is 0.0024 for central nervous system HB and the risk of hemorrhage was determined by the size of the tumors [6]. In our patient, the size of the tumor was relatively large (70*57*60 mm). Similar to cases of arteriovenous malformation, high blood flow through the tumor combined with partial transmission of the arterial pressure to the venous side eventually lead to vascular vulnerability. Moreover, the superficial location of parenchyma without the surrounding support can increase the pressure difference between the inside and outside of the blood vessels, which also increases the risk of hemorrhage. This may explain why most HB that hemorrhage are located at subarachnoid spaces or superficial sites of parenchyma [7]. Clinically, they are usually revealed by signs of intracranial hypertension, as was the case in our patient. In anatomopathology, four forms of HB are described: the simple cyst, the cyst with a wall nodule, the dense tumour and the solid tumour with small internal cysts. This concept is important because the imaging appearance of these lesion forms is directly correlated with their histological constitution [8]. On imaging, the appearance of the tumour depends on its composition. CT scan is usually the first examination performed [9], as was the case in our patient, but MRI remains the examination of choice for the examination of the posterior brain fossa and its tumours, better specifying their cystic and solid components. The injection of gadolinium also makes it possible to detect nodules not revealed by CT scan [10]. The main differential diagnosis of HB is pilocytic astrocytoma. Indeed, these two tumours may have the same characteristics in conventional MRI sequences, especially in cystic forms with wall nodule, hence the interest of infusion sequences, which allow to direct to the right diagnosis before formal confirmation by histological evidence [11]. Indeed, in our patient the diagnosis of a pylocytic astrocytoma was initially made based on the appearance of the tumour on cerebral MRI and the true diagnosis of a cerebellar hemangioblastoma was confirmed only after an anatomopathological analysis of the surgical specimen. The current HB´s management includes surgery, endovascular embolization, radiosurgery and radiotherapy. Complete tumor removal of HB is curative and is associated with low morbidity and mortality (2%) [12]. Radiation therapy is indicated for recurrent HB, partial tumor resection or contraindication to the surgery. In our patient, radiotherapy was an alternative in front of partial surgical ablation, and the evolution was spectacular under this therapeutic weapon, which was administered with good tolerance.

## Conclusion

Our case is an example of HB which is quite rare since it is sporadic and does not fit into the framework of the VHL disease unlike the majority of cerebellar HB which occurs most often in the framework of the VHL syndrome. The result of radiotherapy treatment in cases where surgery is not possible is encouraging.
